# Simulating the enzymes of ganglioside biosynthesis with Glycologue

**DOI:** 10.3762/bjoc.17.64

**Published:** 2021-03-23

**Authors:** Andrew G McDonald, Gavin P Davey

**Affiliations:** 1 School of Biochemistry and Immunology, Trinity College Dublin, Dublin 2, Ireland

**Keywords:** gangliosides, Glycologue, glycosyltransferases, neuropathy, Svennerholm nomenclature

## Abstract

Gangliosides are an important class of sialylated glycosphingolipids linked to ceramide that are a component of the mammalian cell surface, especially those of the central nervous system, where they function in intercellular recognition and communication. We describe an in silico method for determining the metabolic pathways leading to the most common gangliosides, based on the known enzymes of their biosynthesis. A network of 41 glycolipids is produced by the actions of the 10 enzymes included in the model. The different ganglioside nomenclature systems in common use are compared and a systematic variant of the widely used Svennerholm nomenclature is described. Knockouts of specific enzyme activities are used to simulate congenital defects in ganglioside biosynthesis, and altered ganglioside status in cancer, and the effects on network structure are predicted. The simulator is available at the Glycologue website, https://glycologue.org/.

## Introduction

**Figure 1:**
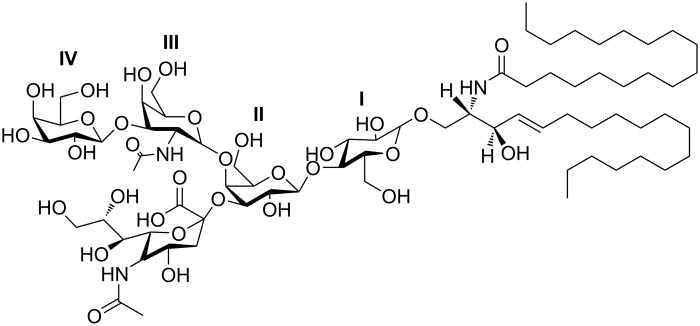
Chemical structure of ganglioside GM1a (a β-ᴅ-galactosyl-(1→3)-*N*-acetyl-β-ᴅ-galactosaminyl-(1→4)-[α-*N*-acetylneuraminyl-(2→3)]-β-ᴅ-galactosyl-(1→4)-β-ᴅ-glucosyl-(1↔1)-ceramide). Substituents of the core are labelled with Roman numerals **I**–**IV** (**I**, Glc; **II**, Gal; **III**, GalNAc; **IV**, Gal). IUPAC name: II^3^Neu5Ac-Gg_4_Cer.

Gangliosides are glycosphingolipids that contain a sialylated carbohydrate linked to ceramide. Typically located in the plasma membranes of many tissues, gangliosides are most concentrated in the brain, where they are the dominant feature of the neuronal glycocalyx [1–3]. The oligosaccharide is based on a linear chain comprising of up to four monosaccharide units, containing glucose, galactose and *N*-acetylgalactosamine, to which are attached a variable number of sialic acid (*N*-acetylneuraminic acid) residues. The sialic acid content of the oligosaccharide, being anionic at pH 7, results in an overall negative charge. Figure 1 shows the structure of the monosialylated ganglioside GM1a.

The biosynthesis of gangliosides occurs in the endoplasmic reticulum and Golgi, where specific glycosyltransferases act, in stepwise fashion, by adding monosaccharides from sugar nucleotide donors, first to ceramide, and then to subsequent ceramide-linked glycoconjugate acceptors, before transport and eventual incorporation into the plasma membrane via vesicular fusion. Gangliosides, which function as antigenic determinants [4], may play a role in membrane organization [5], cell signaling [6], apoptosis [7], and in memory formation through neuromodulation of synaptic transmission [8]. Gangliosides are recycled in the lysosome through the action of glycohydrolases. The inhibition of membrane recycling has been shown to lead to an accumulation of lysosomal gangliosides resulting in neuronal death [9]. Congenital disorders of ganglioside biosynthesis can lead to a number of neuropathies, including motor deficits, microcephaly, sensory loss, and autistic features [10–11]. Certain gangliosides, such as GM2, have been identified as tumor markers for breast cancer stem cells [12], while members of the alpha-series gangliosides, such as GD1α, promote tumor-cell adhesion during metastasis [13]. The cholinergic neuron-specific gangliosides GQ1bα and GT1aα may contribute to the pathogenesis of Alzheimer’s disease [14].

Previously, we described a deductive apparatus of a formal system for modelling the enzymes of mucin-type O-linked glycosylation, with a web-based application, O-Glycologue, that allows knockouts of enzymes of O-linked glycosylation and the assignment of custom “wild type” sets of enzyme activities to study the effects of differential knockouts on the resultant networks [15]. In this article, we describe an extension of this method to gangliosides, and to the enzyme reactions associated with their biosynthesis. The formalism and the associated web application, now renamed Glycologue, provide a way to explore the effects of mutations that result in a loss of functionality, or promotion of disease.

The method involves a set of regular-expression-based rules acting on strings of characters that representing the monosaccharide units, x, model the actions of transferases in the general form, Ax + B = A + xB, where Ax is a nucleotide sugar and B is the carbohydrate moiety of the acceptor, be it a glycolipid or some other oligosaccharide, the nucleotide A is the product of the donor and xB is the acceptor product. The strings can be seen as a compression of the familiar condensed linear IUPAC notation, using a single-letter notation to represent sugars, with upper-case denoting ᴅ, and lowercase, the ʟ, sugars, and are read from right to left starting with the base (reducing-end) sugar. The letters a and b are reserved for α and β-anomers, respectively, while brackets are used to delimit branches, and the letter T is used to denote the connection point to ceramide, or to another conjugate depending on the context. In this work, we consider only four monosaccharides and their corresponding letters: Glc (G), Gal (L), Neu5Ac (S) and GalNAc (V). In IUPAC form (see Table 1), we can write the carbohydrate portion of the ganglioside GM1a (Figure 1) as any of the following:

- Galb1-3GalNAcb1-4[Neu5Aca2-3]Galb1-4GlcCer (IUPAC)
- Lb3Vb4[Sa3]Lb4GbT (Glycologue, full)
- L3Vb4[S3]L4GT (Glycologue, abbreviated)

**Table 1:** Single-letter codes used and their IUPAC equivalents.

Glycologue single-letter code	IUPAC symbol	IUPAC definition

G	Glc	β-ᴅ-glucose
L	Gal	β-ᴅ-galactose
S	Neu5Ac	*N*-acetylneuraminate
T	Cer	ceramide (*N*-acylsphingosine)
V	GalNAc	*N*-acetyl-α-ᴅ-galactosamine
a, b	α, β	anomeric configuration

Glycologue makes the further assumption that each sugar as it appears in the acceptor has a default anomer, with Glc and Gal being β, and Neu5Ac and GalNAc being α, which allows the a and b notation to be dropped in most instances. However, since GalNAc appears as the β anomer in gangliosides, the b is retained in the abbreviated Glycologue notation for any substrate in which it appears. The resulting string is referred to a *structure identifier* [15], since it also contains the instructions for drawing a 2-dimensional image of the oligosaccharide, in the manner of turtle graphics.

## Results and Discussion

### Model description

The simulator acts iteratively on an initial acceptor substrate, passing it to each enzyme in turn, and accumulating a set of acceptor products. The pool of novel acceptor products become the substrates at the next iteration, until either no new products are formed, or a user-determined maximum number of iterations has been reached. Table 2 lists the enzymes of ganglioside biosynthesis included in the current model, with an index number, **1**–**10**, the EC number, where available, a short name, a longer accepted name and a reaction pattern. The reactions in Table 2 are based on activities of enzymes already classified within the IUBMB Enzyme List, or from the cited references, wherever an EC number is not available. Glycosyltransferases can act on a variety of substrates, and in cases where substrate recognition follows a less specific rule, the reaction pattern uses an asterisk to denote parts of the acceptor that are of indeterminate length. The glossary in Table 2, footnote b, shows some of the assumptions implicit to the model, such as the configuration of the donors. From this information it is possible to infer that all of the enzymes of the model are all configuration-inverting, rather than configuration-retaining. This inversion of configuration refers to the stereochemistry of the anomeric carbon in the acceptor product, which has the opposite configuration to that of the donor substrate.

**Table 2:** Enzymes of ganglioside biosynthesis and Glycologue reaction patterns.

enzyme no.	EC number	short name	accepted name^a^	reaction pattern^b^

**1**	EC 2.4.1.80	UGCG	ceramide glucosyltransferase	UDP-G + T = UDP + GbT
**2**	EC 2.4.1.47	β1Gal-T3	*N*-acylsphingosine galactosyltransferase	UDP-L + T = UDP + LbT
**3**	EC 2.4.1.274	β4Gal-T6	glucosylceramide β-1,4-galactosyltransferase	UDP-L + GbT = UDP + Lb4GbT
**4**	EC 2.4.99.9	ST3Gal-V	lactosylceramide α-2,3-sialyltransferase	CMP-S + Lb4GbT = CMP + [Sa3]Lb4GbT CMP-S + LbT = CMP + [Sa3]LbT
**5**	EC 2.4.99.8	ST8Sia-I	α-*N*-acetylneuraminate α-2,8-sialyltransferase	CMP-S + [Sa3]Lb4*T = CMP + [Sa8Sa3]Lb4*T
**6**	EC 2.4.99.- [16]	ST8Sia-V	(α-2,8-*N*-acetylneuraminate α-2,8-sialyltransferase)	CMP-S + [Sa8Sa3]*T = CMP + [Sa8Sa8Sa3]*T CMP-S + Sa8Sa3*T = CMP + Sa8Sa8Sa3*T
**7**	EC 2.4.1.92	β4GalNAc-T1	(*N*-acetylneuraminyl)-galactosylglucosylceramide *N*-acetylgalactosaminyltransferase	UDP-V + Lb4*T = UDP + Vb4Lb4*T UDP-V + [Sa3]Lb4*T = UDP + Vb4[Sa3]Lb4*T
**8**	EC 2.4.1.68	β3Gal-T4	ganglioside galactosyltransferase	UDP-L + Vb4*T = UDP + Lb3Vb4*T
**9**	EC 2.4.99.- [17–18]	ST3Gal-II	(β-1,3-galactosyl-ceramide α-2,3-sialyltransferase)	CMP-S + Lb3Vb4*T = CMP + Sa3Lb3Vb4*T
**10**	EC 2.4.99.- [19]	ST6GalNAc-V	(α1,3-Sia-β1,3-Gal-β1,3-GalNAc α-2,6-sialyltransferase)	CMP-S + Sa3Lb3Vb4*T = CMP + Sa3Lb3[Sa6]Vb4*T

^a^For enzymes without an EC number, a suggested name is given in parentheses. Literature references supporting the unclassified activities are provided in the EC number column, after the EC sub-subclass. ^b^Asterisks act as a wildcard character, to denote an unspecified portion of the oligosaccharide. Symbols and abbreviations used in reaction patterns are those of Table 1 with the following additions: UDP, uridine 5′-diphosphate; CMP, cytidine 5′-phosphate; CMP-S, CMP-*N*-acetyl-β-neuraminate; UDP-G, UDP-α-ᴅ-glucose; UDP-L, UDP-α-ᴅ-galactose; UDP-V, UDP-*N*-acetyl-α-ᴅ-galactosamine.

The formal language on which the method is based is a modelling language for glycosyltransferases, and can be used to classify the types of reaction catalyzed according to simple rules. We identify *extension* of a linear oligosaccharide as the default mode of action (Equation 1),

[1] Ax+yB=xyB+A

where x and y are monosaccharides, Ax is the nucleotide-sugar donor, and yB the acceptor substrate. The formation of a single branch along a linear chain is described as *decoration*, where the pattern is (Equation 2).

[2] Ax+yB=[x]yB+A

Here we have assumed that [x]y is a substring of the parent acceptor and is a shorthand for *[x]y*B, the asterisks acting as a wildcard character. Double branches are used to form symmetric core structures, such as the trimannosyl core of N-glycans, or O-linked glycan cores based on GalNAc (Equation 3):

[3] Ax+yB=[x][y]B+A

Capping of branches and linearly extended chains is achieved through *termination*, of which sialylation is a typical example. However, some enzymes can continue to act on such terminal elements, which can be called *termination with extension*, or *decoration with extension*. As an example of the latter, LacCer (L4GT) can be decorated on the galactose by enzyme **4** to give [S3]L4GT, and subsequently extended by enzymes **5** and **6** to give [S8S3]L4GT and [S8S8S3]L4GT. Termination with extension in this model occurs at the initial sialylation of L3Vb4L4GT by enzyme **9**, to yield S3L3Vb4L4GT, which can be further extended by two iterations of **6**, to produce S8S8S3L3Vb4L4GT. A separate category not considered in this model is *modification* of monosaccharides, for example through sulfation, acetylation or phosphorylation, which follow the same pattern as decoration. Glycologue structure identifiers order branches by linkage position, writing the branch with the lowest linkage first, reading from right to left. Modifiers are written before sugars units, and multiple modifiers on the same monosaccharide are again ordered by linkage position, from lowest to highest, reading right to left.

### Nomenclature of gangliosides

Gangliosides are commonly labelled according to the abbreviated Svennerholm [20] nomenclature, or else by the expanded form recommended by IUPAC/IUBMB Joint Commission on Biochemical Nomenclature [21]. The original Svennerholm notation was a semi-systematic system, and its formation rules have not always consistently applied by those using it. We introduce here a more systematic Svennerholm nomenclature that reproduces, as far as possible, the traditional system, but which is capable of automatic assignment by Glycologue from the structural identifier, and then translation to the IUPAC form. A description of the method will be given, together with examples.

In the IUPAC system, ganglio-series of glycosphingolipids are given the core abbreviation, Gg, followed by the number of monosaccharides (-oses) in the linear core, as a subscript. Thus, the core descriptor Gg*_n_*Cer represents a core of length *n* attached to ceramide. (Formerly, the core descriptor was given as “GgOse*_n_*Cer” [22], but this recommendation has since been rescinded [21].) To this base string are added a list of the sialic acids attached to each monosaccharide in the core, counting using the Roman numeral system, starting from the base glucose (cf. Figure 1). From the non-reducing end, write the position on the core where a sialic acid (or sialic acid chain) appears, as the uppercase Roman numeral, superscripting the linkage position after as the Arabic numeral, followed by “Neu5Ac”; if a chain of sialic acids is present, place the “Neu5Ac” in parentheses and subscript the number of residues after, e.g., IV^3^(Neu5Ac)_2_. Repeat this procedure for as many units of the core as are sialylated, separating with commas, then append a hyphen, followed by the core descriptor.

The systematic Svennerholm nomenclature system used by Glycologue counts the total number of sialic acids in the carbohydrate, and appends this as a single letter (A: Asialo = 0, M: Monosialo = 1, D: Disialo = 2, T: Tri = 3, etc.) to the “G” of ganglioside. The core is assigned a number based on its length, where 1 denotes fully extended, Galb1-3GalNAcb1-4Galb1-4GlcCer, 2 is GalNAcb1-4Galb1-4GlcCer and 3 is Galb1-4GlcCer. When the core is fully extended, the series letter, a, b or c, is appended, which denotes the presence of either one, two or three sialic acids on position II of the core. The presence of sialylation on the root galactose of LacCer thus determines the series into which the ganglioside is categorized. A common practice, although not recommended by IUPAC, is to add α at the end of the code, when an α-2,6-linked Neu5Ac is present on position III, which is the GalNAc β4-linked to Gal. The composition of the systematic Svennerholm name (SSN) is then

“G” + (the total sialic-acid count, as a capital letter) + (4 – *n* + 1) + (series letter a–c, where *n* = 4) + (α),

where *n* is the core length as defined above. GalCer is denoted by the core number 4, and hence is synonymous with GA4, while its monosialylated form is denoted GM4.

**Figure 2:**
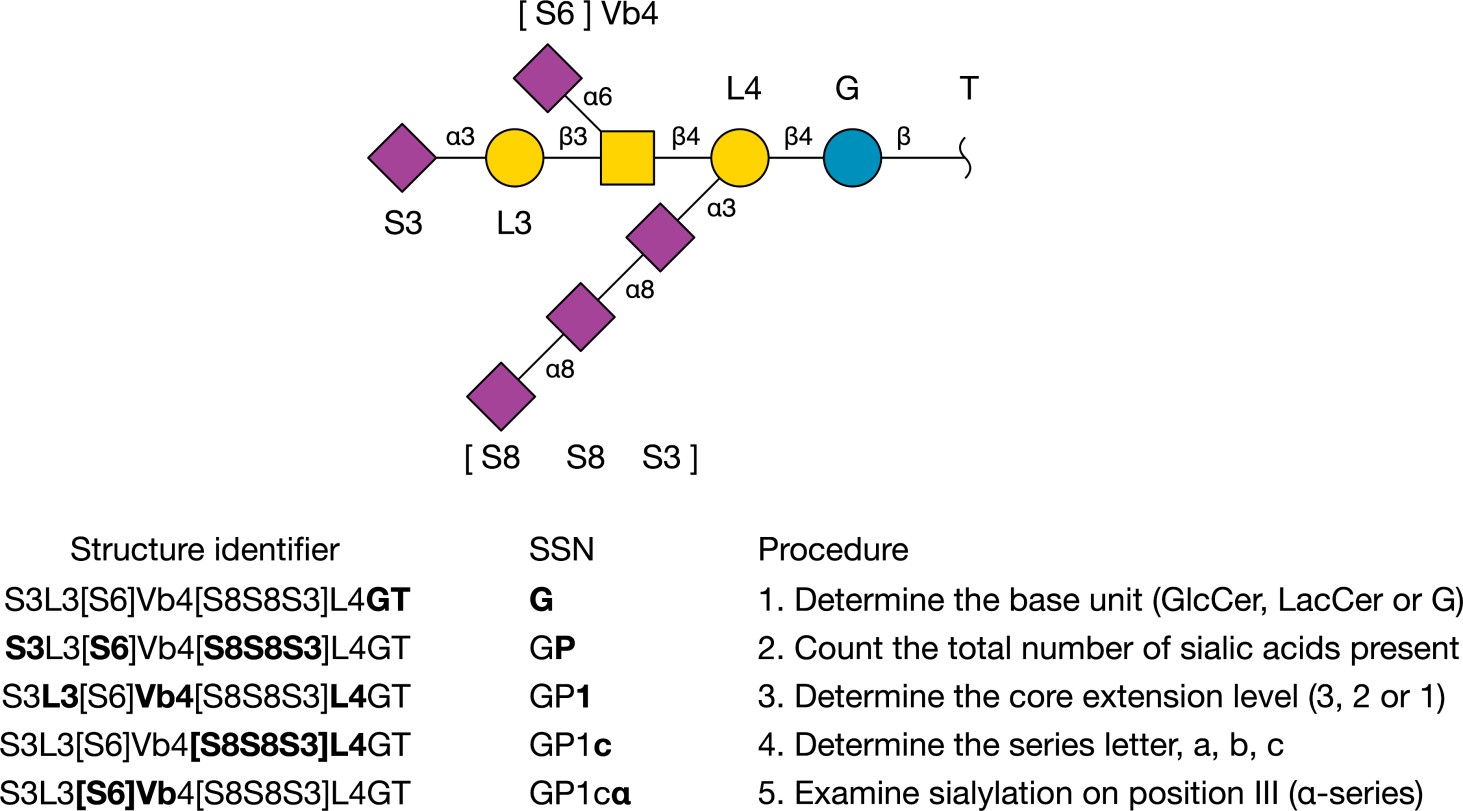
Construction of the Svennerholm name GP1cα from its Glycologue structure identifier. At each step of the procedure, the parts of the structure identifier that determine the corresponding part of the systematic Svennerholm name (SSN) are shown in bold face.

To illustrate the method with an example (see Figure 2), the simulator predicts the existence of the ganglioside carbohydrate with structure identifier S3L3[S6]Vb4[S8S8S3]L4GT. The total sialic acid count of this structure is 5, which is assigned the letter P (penta-sialylated). It is a c-series ganglioside, with 3 sialic acid residues on position II. There is 1 sialic acid attached to the GalNAc, which means that there must be one (5–3–1) sialic acid on the terminal non-reducing Gal (position IV). The core is fully extended, which gives *n* = 4, therefore the SSN is GP1cα.

The IUBMB name follows from the systematic Svennerholm code. The fact that there are five (P) sialic acids, three of which are on position II (c), and one on position III (α), implies that the core must be fully extended, with the remaining sialic acid on position IV. The IUPAC name of this structure is therefore IV^3^Neu5Ac,III^6^Neu5Ac,II^3^(Neu5Ac)_3_-Gg_4_Cer.

**Figure 3:**
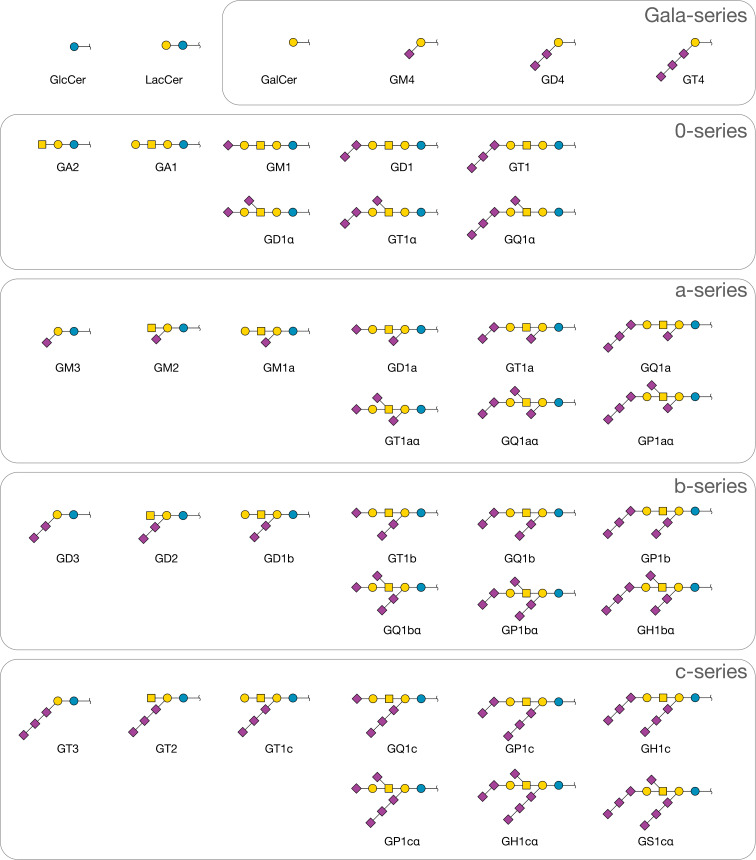
Ganglioside carbohydrates predicted by the model. All structures are linked to ceramide at the base glucose or galactose residue.

The SSN is identical to that which is generally used, with the exceptions noted in Table 3. It should be noted that the name “GM1” becomes ambiguous if the letter a–c is not consistently applied when the core is fully extended (*n* = 4), since there are two galactose residues (positions II and IV) at which sialylation can occur. The name “GM1b”, which has been used to refer to GM1 (IV^3^Neu5Ac-Gg_4_Cer) is formally incorrect, since it is not a b-series ganglioside, with two Neu5Ac residues on position I, but in the 0-series gangliosides that derive from GA2 (Figure 3).

**Table 3:** Commonly used Svennerholm names that differ from their systematic counterparts.

Svennerholm name (incorrect)	systematic Svennerholm name (SSN)	IUPAC name

GM1b	GM1	IV^3^Neu5Ac-Gg_4_Cer
GM1	GM1a	II^3^Neu5Ac-Gg_4_Cer
GD1c	GD1	IV^3^(Neu5Ac)_2_-Gg_4_Cer
GD1aα	GD1α	IV^3^Neu5Ac,III^6^Neu5Ac-Gg_4_Cer

### Enzymes of the model

**Figure 4:**
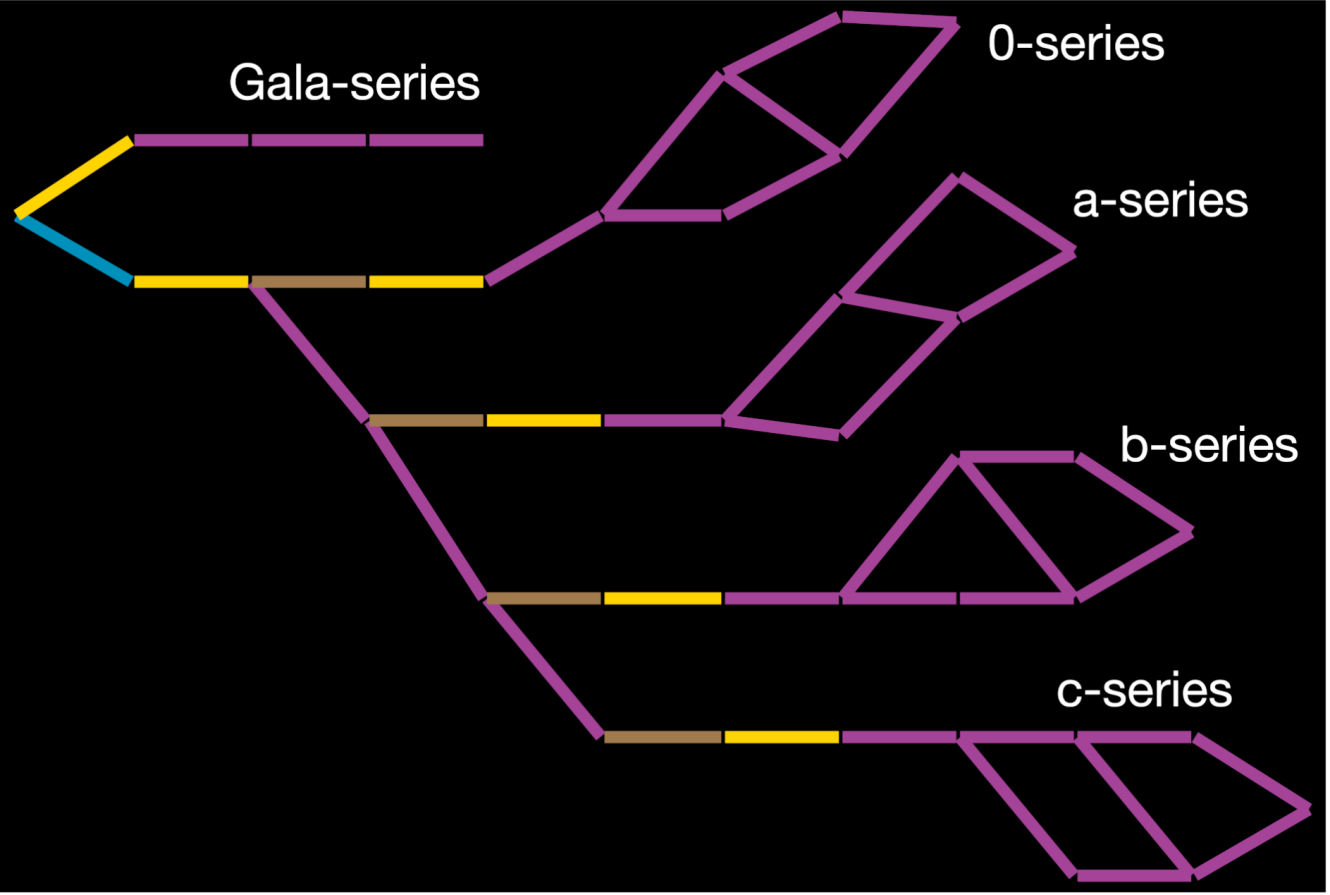
Ganglioside biosynthetic reaction network predicted by the Glycologue enzyme simulator. Starting from ceramide, which is the root (leftmost) node, 41 carbohydrate structures are predicted using 10 enzymes. The edges of the graph represent enzyme reactions, colored according to the type of sugar transferred: yellow (galactosyltransferases); blue (glucosyltransferases); brown (*N*-acetylgalactosaminyltransferases); magenta (sialyltransferases).

Using the preceding classification, we see that the enzymes of Table 2 fall into five categories: extension (activities **1**–**3**, **5**, **7**, and **8**), decoration with extension (**4**), decoration (**10**), termination with extension (**9**), and termination (**6**), all of which follow the reaction patterns of Equation 1 and Equation 2, and none follow the double-branching pattern of Equation 3. The first iteration, starting from ceramide (T), produces GlcCer, catalyzed by ceramide glucosyltransferase (EC 2.4.1.80). Also included is the activity of *N*-acylsphingosine galactosyltransferase (EC 2.4.1.47), which produces GalCer, the starting member of the Gala series of galactocerebrosides. GlcCer, but not GalCer, can at the next iteration be extended with a galactose, β4-linked to the glucose, to form lactosylceramide, LacCer, catalyzed by β4Gal-T6 (EC 2.4.1.274). This is the first asialo-ganglioside, with core level 3. Two further extensions are possible, to yield core levels 2 and 1, by adding a GalNAc residue β4-linked to the preceding galactose (EC 2.4.1.92), followed by a further galactose in a β3-linkage to GalNAc. The maximally extended core oligosaccharide is thus L3Vb4L4GT in the abbreviated Glycologue notation. Sialylation can occur in the model through decoration of the base galactose, or termination of the β3-linked galactose, by the α-2,3-sialyltransferase enzymes ST3Gal-V (**4**) and ST3Gal-II (**9**), also known as GM3 synthase and GM1 synthase [23–24], respectively. It is assumed that the sialyltransferase activity **5** occurs before core extension with GalNAc by EC 2.4.1.92 (**7**). Up to two further sialylation steps can occur, on each of the sialylated galactose positions, with α-2,8-linkage (activities **5** and **6**). Ganglioside GM1 can act as the substrate of ST6GalNAc-V (**10**), which adds a α-2,6-linked Neu5Ac to the central GalNAc residue of the core. As it is known that certain isoforms of ST3Gal-V can act on GalCer [25–26], this alternative activity of the enzyme has been included in the model (Table 2). After 11 iterations of the method, the simulator produces 41 unique structures shown in Figure 3, in 49 reactions, with the network shown in Figure 4.

### Networks and knockouts

The structures shown in Figure 3 are divided into five subsets: the Gala series, 0-series, a-series, b-series, and c-series gangliosides. Starting from GalCer (GA4), the model predicts three downstream products, GM4, GD4, and GT4, through the sequential action of ST3Gal-V (**4**) and two applications of the enzyme ST8Sia-I (**5**). These structures have previously been observed in bovine milk [27]. The 0-series gangliosides produced, in addition to asialo-gangliosides, the sialylated forms GM1, GD1, and GT1, along with their α2-,6-sialylated counterparts, GD1α, GT1α, and GQ1α. Also in Figure 3 are the a-series gangliosides GM3, GM2, and GM1a, and the derivatives of the GM1a with terminal sialylation, GD1a, GT1a [28], and GQ1a. The b-series gangliosides, GD1b, GT1b (both downregulated in Alzheimer’s disease [14,29]), GQ1b and GP1b, and their three α-variants are predicted. There is no GT1bα, since a terminal sialic acid on position IV is required by enzyme rule **10**. The c-series gangliosides follow the same pattern as those of the b series. The members of this series with fully extended core (GT1c, GQ1c, GP1c and GH1c) appear in stellate neurons of adult human brain [30], but are also found in extraneural tissues in species such as rat [31]. The model predicts the structures of GP1cα, GH1cα and GS1cα, which may correspond to the penta-, hexa-, and septa-sialylated gangliosides observed in embryonic chicken brain [32].

**Figure 5:**
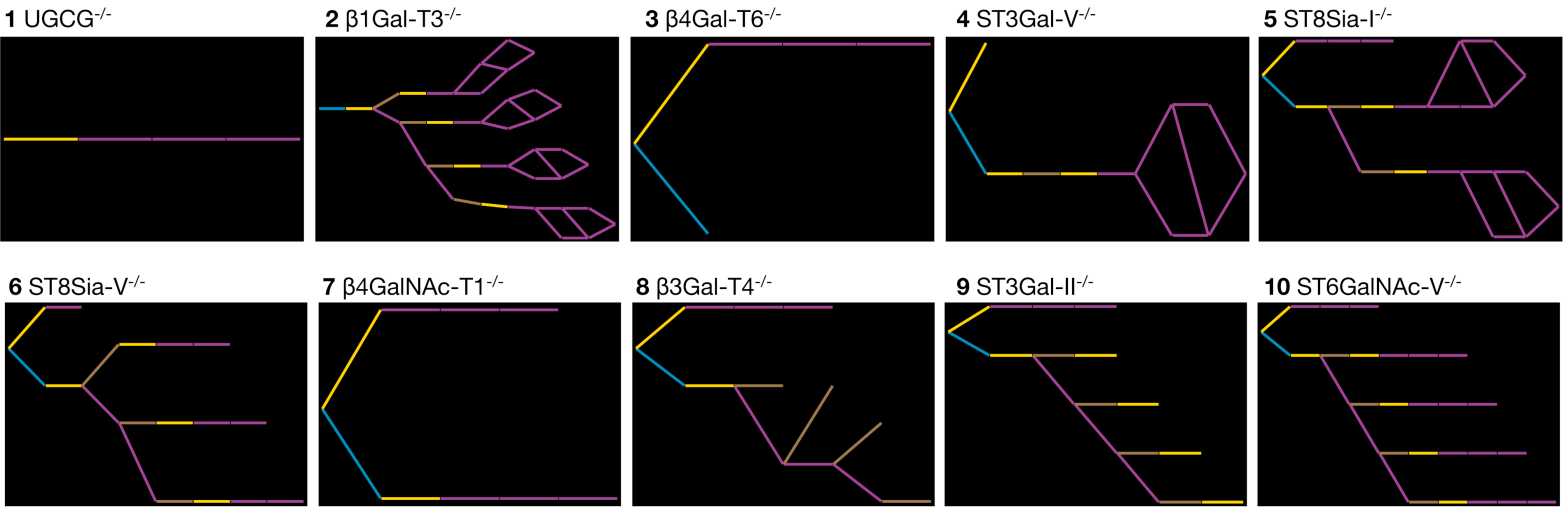
Predicted effects on the pathways of ganglioside biosynthesis when individual enzyme activities are completely inhibited or knocked-out. Panels 1–10 correspond to the enzymes of Table 2. Enzyme reactions are shown as lines colored according to the type of sugar transferred: yellow (galactosyltransferases); blue (glucosyltransferases); brown (*N*-acetylgalactosaminyltransferases).

The effects of knocking out each enzyme of Table 2 individually are shown in Figure 5. Comparing the pattern of glucosylation, sialylation, galactosylation, and GlcNAc-ylation events among the different knockouts, and with that of the full network in Figure 4, reveals that the most pronounced effects on ganglioside complexity occur with enzyme activities **1**, **3**, and **7**, which result in fewer than 10 reactions each. That any structures are formed in the absence of UGCG (**1**) is because of the Gala structures formed by *N*-acylsphingosine galactosyltransferase (**2**). Mutations in the gene coding for enzyme **7** (β4GalNAc-T1; also known as GM2/GD2 synthase [33]) are responsible for spastic paraplegia [10]. The knockouts affecting entire series, discounting those of Gala, are the enzymes **4**, **5**, and **6**, which produce only the 0-series (**4**), 0- and a-series (**5**) and 0-, a-, and b-series gangliosides (**6**). The loss of GM3 through ST3Gal-V (**4**) deficiency is associated with auditory impairment in mouse and human [34]. The loss of complex polysialylated structures is evident in the knockouts of enzyme activities **8** and **9**, which are unable to form terminal sialic acid or α-type structures. Knockout of ST3Gal-II (**9**) reduces GD1and GT1b levels in the brain by 50%, whereas brain-protein sialylation is unchanged [35]. A loss of ST3Gal-II also leads to late-onset obesity and insulin resistance [36].

### Glycologue web application

The Glycologue ganglioside simulator is available at https://glycologue.org/g/, along with the source code of the simulator in the Python programming language. Glycologue exports networks as SBML, for import into Copasi [37], CellDesigner [38], Tellurium [39], or other modelling software supporting this format. Glycan structures can be imported or exported as GlycoCT [40], and exported as Linear Code [41] or IUPAC condensed linear formats. Sets of structures can be downloaded as CSV or GlycoCT. A key function of Glycologue is the ability to predict the enzymes required for the biosynthesis of a given glycan; the subset of the enzyme activities can then be used to generate all of the ganglioside carbohydrates, starting from ceramide, or any other structure. By setting a baseline knockout of enzyme activities, the effects of further knockouts on the number and type of glycans formed can be predicted. In the web application version of Table 2, reactants and reactions are linked to ChEBI [42] and Rhea [43] by their identifiers, where these are available.

#### Future development

In addition to biosynthesis, the biochemistry of gangliosides includes cellular transport and recycling. A limitation of the model is that it considers only absolute changes to enzyme activity, in which an activity is either off or on, which a kinetic model based on differential-equation-based rate laws [44–48] or stochastic kinetics [49–50] would improve upon. Nevertheless, we have shown that the knockouts are able to reproduce the distinct species-specific features and disease states arising from congenital defects of ganglioside biosynthesis. Kinetic models based on the networks described here can be generated in modelling software, using the SBML output provided. In such models, we suppose that, owing to the multi-branched structure of the networks (cf. Figure 4), multi-substrate competition effects would need to be taken into account [44], since multiple substrates compete for the same enzyme. Competing fluxes downstream of branch points will also influence the kinetics [48,51]. Future extensions to this work will consider the effects of acetylation of sialic acid residues, since this modification reduces the negative charge of the carbohydrate, thus altering binding affinity, while an increased incidence of 9-*O*-acetylated GD3 is associated with melanoma [52]. The activities of glycosidases might be added to the simulators as a way to model lysosomal storage diseases (LSDs) such as Tay-Sachs, in which ganglioside GM2 accumulates as a result of a deficiency in β-*N*-acetylhexosaminidase activity (EC 3.2.1.52) [53]. Since Glycologue structure identifiers can be exported as Linear Code, future support for the recently introduced LiCoRR (Linear Code for Reaction Rules) formalism [54] is also possible. Glycologue can incorporate the Neu5Gc and KDN variants of sialic acid, and predict structures containing these residues. However, they have not been considered here, from a de novo standpoint, owing to the combinatorial complexity that would arise from equal participation of the donors CMP-Neu5Ac, CMP-Neu5Gc, and CMP-KDN.
